# Understory plant biodiversity is inversely related to carbon storage in a high carbon ecosystem

**DOI:** 10.1002/ece3.70095

**Published:** 2024-10-27

**Authors:** Trevor A. Carter, Brian Buma

**Affiliations:** ^1^ Department of Forest and Rangeland Stewardship Colorado State University Fort Collins Colorado USA; ^2^ Environmental Defense Fund Boulder Colorado USA

**Keywords:** co‐management, forest biodiversity, forest carbon, plant species diversity, plant species richness, tree biomass carbon

## Abstract

Given that terrestrial ecosystems globally are facing the loss of biodiversity from land use conversion, invasive species, and climate change, effective management requires a better understanding of the drivers and correlates of biodiversity. Increasingly, biodiversity is co‐managed with aboveground carbon storage because high biodiversity in animal species is observed to correlate with high aboveground carbon storage. Most previous investigations into the relationship of biodiversity and carbon co‐management do not focus on the biodiversity of the species rich plant kingdom, which may have tradeoffs with carbon storage. To examine the relationships of plant species richness with aboveground tree biomass carbon storage, we used a series of generalized linear models with understory plant species richness and diversity data from the USDA Forest Service Forest Inventory and Analysis dataset and high‐resolution modeled carbon maps for the Tongass National Forest. Functional trait data from the TRY database was used to understand the potential mechanisms that drive the response of understory plants. Understory species richness and community weighted mean leaf dry matter content decreased along an increasing gradient of tree biomass carbon storage, but understory diversity, community weighted mean specific leaf area, and plant height at maturity did not. Leaf dry matter content had little variance at the community level. The decline of understory plant species richness but not diversity to increases in aboveground biomass carbon storage suggests that rare species are excluded in aboveground biomass carbon dense areas. These decreases in understory species richness reflect a tradeoff between the understory plant community and aboveground carbon storage. The mechanisms that are associated with observed plant communities along a gradient of biomass carbon storage in this forest suggest that slower‐growing plant strategies are less effective in the presence of high biomass carbon dense trees in the overstory.

## Graphical abstract

Understory plant species richness and plant community diversity are inversely related to aboveground live tree biomass carbon. On average, there is a decline of 2 plant species per increase in 50 Mg ha^−1^ aboveground live tree biomass. There is an apparent tradeoff between plant biodiversity and carbon management.

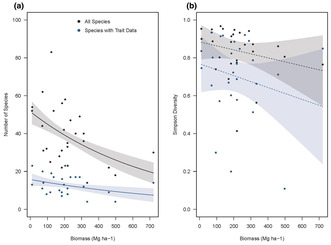

## 1 INTRODUCTION

Terrestrial ecosystems globally are facing the loss of biodiversity from land use conversion, invasive species, and climate change (Caro et al., 2022; Heller & Zavaleta, 2009), but effective management requires a better understanding of the drivers and correlates of biodiversity. Understanding the relationship between species diversity and abundances with other ecosystem functions/services is increasingly important as global climates change (Thom & Seidl, 2016). Biodiversity is often positively related to the effectiveness, reliability, and diversity of ecosystem services (Vos et al., 2014). However, the Intergovernmental Panel on Climate Change (IPCC) estimates that with a temperature increase of 1.6°C, more than 10% of all species will become endangered (Pörtner et al., 2022). These declines in biodiversity are likely to correspond with degraded habitats and cause decreases in ecosystem services (Pörtner et al., 2022). Biodiversity, however, may be maintained in the context of climate change via focused management strategies that conserve species through the protection of habitat (Heller & Zavaleta, 2009). The protection of species habitat often corresponds with the maintenance, or increase, of carbon stocks in forested landscapes, which is another common management goal in the face of climate change (Paoli et al., 2010).

The co‐management of biodiversity and forest carbon primarily targets the conservation of high carbon trees, which are typically large in diameter (Anderson et al., 2009; Armenteras et al., 2015; Lecina‐Diaz et al., 2018). Large trees hold disproportionally more carbon than smaller trees and provide structural diversity with a range of microhabitats for a variety of vertebrate and invertebrate species (Lecina‐Diaz et al., 2018). Positive correlations between forest carbon storage and species diversity vary in strength depending on the spatial scale under investigation and are strongest when evaluating broader scale trends of carbon storage and vertebrate diversity (Di Marco et al., 2018). However, previous investigations on the co‐management of biodiversity and carbon storage generally do not focus on the biodiversity of the species rich plant kingdom and their potential relationship with carbon. This is a large gap in our understanding of the tradeoffs of carbon management and biodiversity for some of the most taxonomically numerous and otherwise important members of ecosystems not previously considered in biodiversity assessments (Di Marco et al., 2018; Midgley et al., 2010; Soto‐Navarro et al., 2020).

Plants are more taxonomically diverse than other common groups that are researched in biodiversity studies, such as mammals or birds (Enquist et al., 2019). Understory plant species, which are relatively less studied than dominant forest tree species (Whigham, 2004), provide important ecosystem services such as biogeochemical cycling (Misson et al., 2007; Speckman et al., 2015), wildlife habitat and nutrition (Przepióra et al., 2020; Rhoades et al., 2018; Springer et al., 2022), and carbon sequestration (Chen et al., 2019; Speckman et al., 2015). Large and competitive trees that store high levels of biomass carbon are likely in competition with the understory plant community, which suggests a potential tradeoff between carbon storage in overstory trees and biodiversity in the understory (Kobayashi et al., 2023). This is especially important in temperate forest ecosystems that have low species richness and diversity in the overstory but higher species richness or diversity in the understory. The inverse relationship between overstory tree basal area and understory plant biodiversity is well documented in other contexts, such as insect outbreaks (Carter et al., 2022; Pappas et al., 2022) and fire (Laughlin & Fulé, 2008; Stevens et al., 2019). However, the potential mechanisms that control the response of the understory plant community in intact forests likely differ from disturbance contexts because carbon dense overstory trees are acquiring or restricting resources (e.g., light; Mouillot et al., 2013; Andrade et al., 2021), as compared to resources being released through the mortality of overstory species (Boggs Lynch et al., 2021). Thus, in addition to elucidating the relationship of plant biodiversity and biomass carbon storage, understanding the potential mechanisms that limit or promote understory plant species richness and diversity in high biomass carbon forests will be critical for future management.

Plant functional traits, specifically those from the leaf economic spectrum, provide an insight into the potential mechanisms that might promote or constrain biodiversity‐carbon tradeoffs (Westoby et al., 2002; Wright et al., 2004). Species vary in their trait values, which support different strategies for growth and survival (Laughlin et al., 2010). For example, species with more conservative leaf traits such as low specific leaf area (SLA) and high leaf dry matter content (LDMC) may be better equipped to persist in nutrient‐competitive environments (Laughlin et al., 2010; Wright et al., 2004), such as those under large trees. Similarly, species that are on average taller are likely better competitors for available light in a potentially light‐limiting environment (Navas & Violle, 2009). The functional traits present in the understory plant community likely vary along a gradient of overstory competition (i.e., live tree biomass) because high biomass carbon (competitive) forests constrain community members from the broader species pool to those with traits that best accommodate conditions under the large‐tree canopy (Keddy & Laughlin, 2022).

Understanding the relationship between carbon storage, plant biodiversity, and key plant traits in a carbon dense forest addresses the importance of potentially deleterious management tradeoffs—managing for more aboveground biomass carbon storage vs. more plant biodiversity. We contribute to this conversation through two main objectives: (1) quantifying the relationship of plant species richness and diversity along a gradient of aboveground live tree biomass carbon storage and other environmental and climatic gradients; (2) evaluating the support for plant functional traits as predictors of the plant community along a gradient of tree biomass carbon storage in a temperate carbon dense forest. First, we predict that understory plant species richness and diversity will be inversely related to tree carbon storage, such that areas with high tree carbon storage have relatively lower understory plant species richness and diversity. An inverse relationship between understory plant species richness and diversity to tree basal area is well documented in post‐disturbance environments (e.g., Carter et al., 2022 for spruce beetle disturbance, Stevens et al., 2019 for fire). We predict areas with warmer average temperatures, lower elevations, and more southerly aspects will be associated with lower understory plant species richness and diversity on average because these conditions typically favor aboveground tree growth and subsequent carbon storage (Buma & Barrett, 2015). Second, we predict that plant communities with more conservative plant functional traits associated with nutrient utilization (low specific leaf area and high leaf dry matter content) and more acquisitive functional traits for light acquisition will be more prevalent in the competitive environment under the canopy of large, carbon dense trees.

## 2 MATERIALS AND METHODS

**FIGURE 1:**
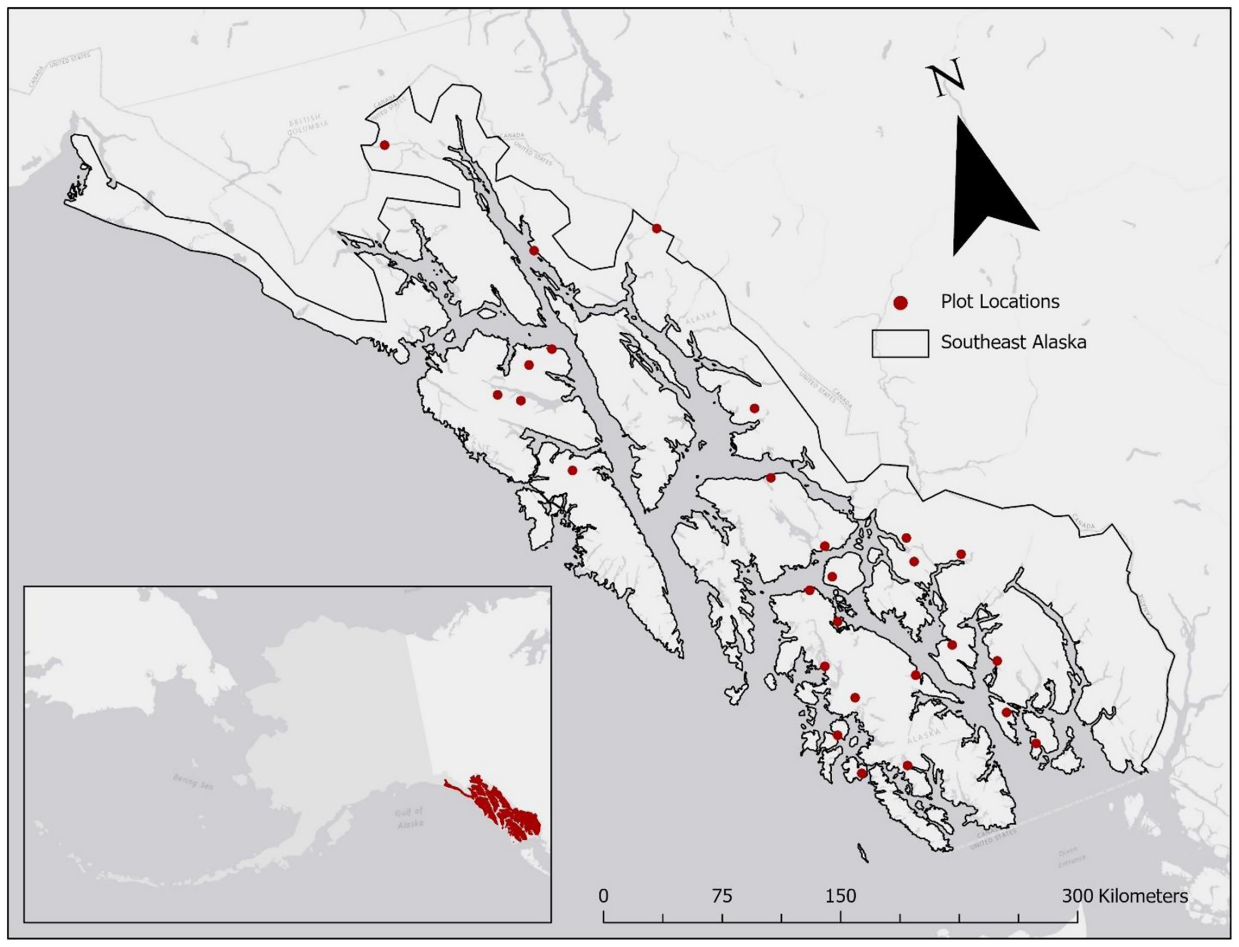
Map of the plot locations (shown in red, *n* = 27) within the Tongass National Forest of southeast Alaska.

To help understand the relationship between plant biodiversity and carbon storage, we utilized understory plant community data and aboveground live tree biomass carbon estimates from the Tongass National Forest (NF) in southeast Alaska. The Tongass NF ranges from 54.6° to 59.9° latitude, occurs within 75 km of the Alaska coast, and thus experiences a cool maritime climate with high annual precipitation (DellaSala, 2011; Figure 1). The Tongass NF is the world's largest intact temperate rainforest and the most biomass carbon‐dense biome type in the world (Keith et al., 2009) with carbon densities ranging from 159 Mg ha^−1^ to 218 Mg ha^−1^ (Carter and Buma *in review*). A few species, *Picea sitchensis, Tsuga heterophylla, Tsuga mertensiana*, and *Callitropsis nootkatensis* dominate the overstory plant community.

We analyzed understory plant community data from the Forest Inventory and Analysis (FIA) permanent sample plot network (Gray et al., 2012). The FIA plot network consists of up to four fixed area (0.0167 ha or 0.0168 ha) subplots that are uniformly stratified across the landscape in forested areas. For this analysis, we averaged plant cover across subplots so that the FIA plot was the experimental unit. Within the Tongass NF, 27 FIA plots provide understory plant data (Figure 1) and high‐resolution modeled carbon data (ranging from 9.71 Mg ha^−1^ – 720 Mg ha^−1^; Carter & Buma, 2024).

We quantified understory plant biodiversity through two different methods. Our first method focused on plant species richness and included a count of understory plant species using 3 different subsets of understory species. Our first subset included all species found in any of the subplots (number of species = 230). Our second subset only included species with associated functional trait data (number of species = 69). Finally, our third subset limited the number of species to only angiosperms and excluded overstory species found in the understory (gymnosperm regeneration), ferns, fern‐allies, and unknowns (number of species = 190). This grouping represents understory species that may be a primary management concern for animal forage and habitat (Hanley et al., 2012) and is presented in the supplement. Our second method of quantifying biodiversity utilized the Simpson diversity index, which incorporates the number of different species present as well as their relative abundances (percent cover within a plot, reported in the FIA data) within each plot for the same three subsets as above.

To investigate the effect of aboveground live tree biomass carbon on understory plant species richness and diversity, we used the modeled estimates of aboveground live tree biomass carbon presented in Carter and Buma (2024), which used the same FIA plots. We used species richness and diversity as response variables and aboveground live tree biomass carbon as a predictor variable in a generalized linear model with a Poisson distributed error structure for species richness or Gaussian error structure for species diversity in an R environment (R Core Team, 2022). For both sets of models, we included additional topographic and climatic covariates that are known to be important for understory species richness and diversity such as elevation (m) and aspect (linear transformed to be degrees from north), as well as precipitation (mm) and temperature (°C; Fick & Hijmans, 2017) to incorporate the partial effects of environmental covariates on the understory. We checked all covariates for collinearity prior to constructing models (Zuur et al., 2010) and repeated this modeling framework for our increasingly stringent subsets of understory plant species (all species vs. angiosperms vs. species with functional trait data). To estimate the change in species (for all subsets) per change in biomass, we ran the model for lowest and highest observed biomass to estimate average associated species change when all coefficients were incorporated.

To further understand the relationship between plant diversity and tree biomass carbon storage in a carbon dense forest, we constructed an ordination. Non‐Metric Multi‐Dimensional Scaling (NMDS) ordinations compress multivariate data, such as understory plant community diversity, into 2 dimensions. Points within the ordination that are closer together are more similar than points that are farther apart (Shipley, 2021). We grouped plots into two categories: (1) either containing aboveground live tree biomass carbon greater than or equal to the 75th percentile (arbitrarily chosen to represent high biomass locations) or (2) less than the 75th percentile of aboveground live tree biomass carbon. We tested the importance of our arbitrary categories for determining group differences by repeating our ordination with more and less stringent categories for what constitutes high biomass (85th and 65th percentile, respectively). We utilized the vegan package to compute our ordination with 1000 permutations (Oksanen et al., 2020).

We incorporated plant functional trait data from the TRY database, a global database of plant functional traits, to better understand the potential mechanisms that promote or constrain understory plant biodiversity (Kattge et al., 2011). Understory functional traits represent major axes of variation in plant strategies such as vegetative height (mm), leaf dry matter content (g/g), and specific leaf area (mm^2^/mg). Specific leaf area measurements excluded petioles when data was available, but in some instances petiole measurements were included or were undefined whether petiole measurements were included Table S1. We were able to calculate species level average trait data for 69 of the 230 species present in the understory FIA dataset (Table S1). These species represent 24% of species cover present across plots and 42% of species cover excluding gymnosperms (overstory species), ferns, fern‐allies, and unknowns. We used the vegan package to create community weighted mean (CWM) trait values, which only incorporate the species with associated trait data for each plot (Oksanen et al., 2020). To further understand the relationship between community weighted mean trait values of height, leaf dry matter content, specific leaf area, and aboveground live tree biomass carbon storage, we utilized generalized linear models with biomass carbon as a predictor variable and community weighted mean trait values as the response variable and a Gaussian error structure.

## 3 RESULTS

**FIGURE 2:**
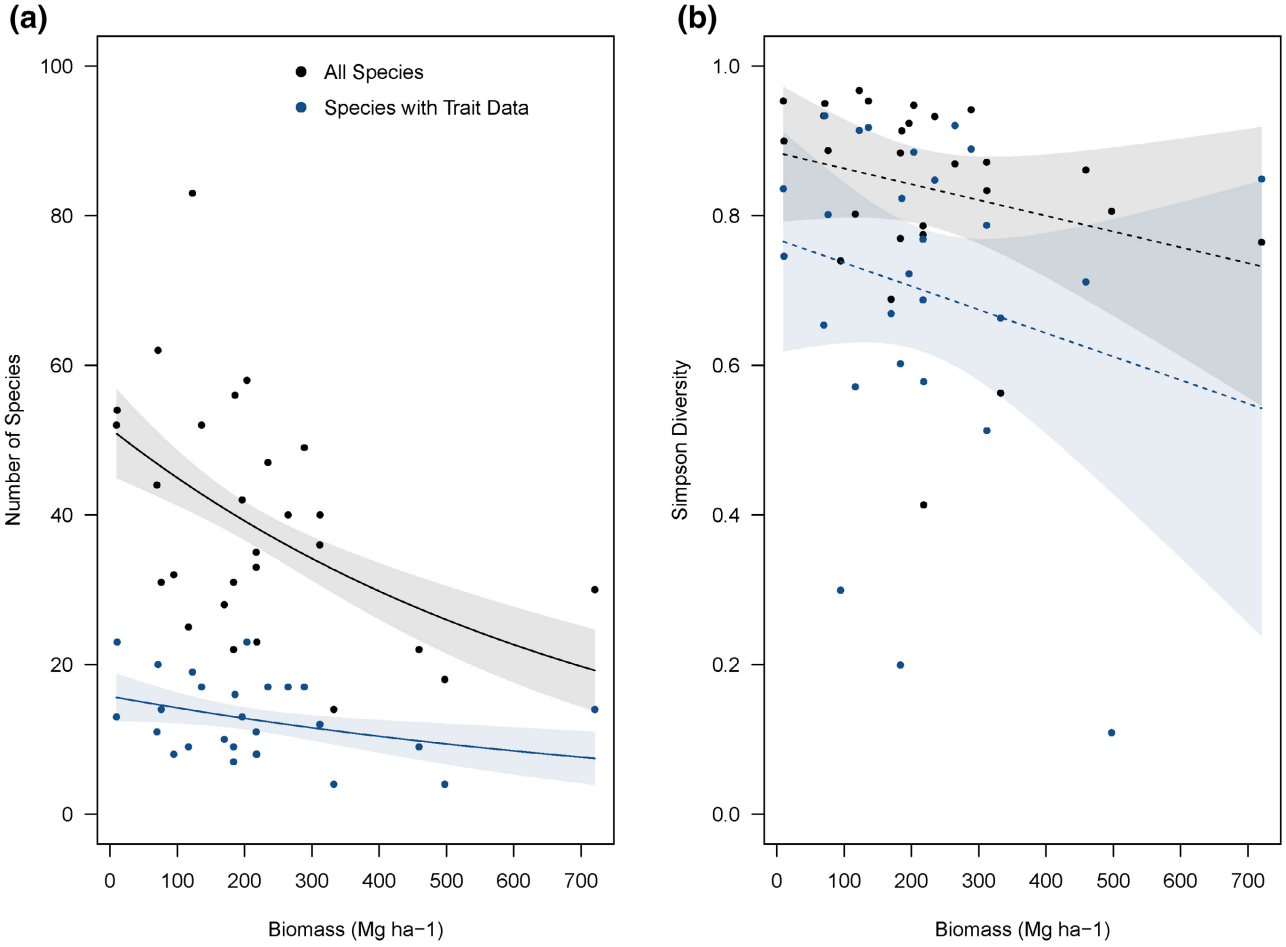
Represents the relationship of species richness (a) and Simpson diversity index (b) as a function of aboveground live tree biomass (Mg of Carbon ha‐1). Solid lines represent statistically significant (alpha = .05) best fit regression lines from our models, while dashed lines represent statistically non‐significant relationships. The shaded polygons represent the 95% confidence interval around the beta estimate for each corresponding slope (see Data S1). The estimated partial effect of live tree biomass carbon on all species found in the understory (black line; 100% of total understory cover) and species with associated trait data (blue line; 23% of total understory cover). An intermediate set of species that includes only angiosperms is presented in Figure S2.

Understory plant species richness declined on average at the plot level as aboveground live tree biomass increased (Figure 2a; Figure S1a); however, the effect size varied by species grouping (Table 1). The model that included all species found in the dataset (Table 1) produced an average decline of 2.23 species (2.19–2.26; 95% CI) per increase in 50 Mg C ha^−1^ with similar declines in the angiosperm only subset (Table S2; decline of 1.95 species [1.86–2.04; 95% CI] per 50 Mg C ha^−1^ increase) and the subset that only included species with associated functional trait data (Table 1; decline of 0.570 species [0.550–0.600; 95% CI] per 50 Mg C ha^−1^ increase). Across all metrics, the decline in species richness was greatest at lower biomass carbon values and became less pronounced at larger biomass carbon values. The Simpson's diversity index, for all subsets, was not related to aboveground live tree biomass (Figure 2b; Figure S1b; Table 1; Table S2).

**TABLE 1:** Parameter estimates (rows) for the effect of the median estimate of aboveground live tree biomass carbon and other known important climatic and topographic predictors on understory species richness and diversity for the all species and species with trait data subsets.

Parameter	All spp. richness (*R* ^*2*^ *= .325*)	Spp. with trait data richness (*R* ^*2*^ *= .205*)	All spp. diversity (*R* ^*2*^ *= .240*)	Spp. with trait data diversity (*R* ^*2*^ *= .283*)
Intercept	**3.54** (1.71 × 10^−1^)	**2.39** (3.03 × 10^−1^)	**7.48 × 10** ^**−1**^ (1.30 × 10^−1^)	2.89 × 10^−1^ (2.12 × 10^−1^)
Elevation (m)	1.63 × 10^−4^ (1.77 × 10^−4^)	−4.50 × 10^−4^ (3.30 × 10^−4^)	1.12 × 10^−5^ (1.43 × 10^−4^)	−2.07 × 10^−4^ (2.33 × 10^−4^)
Aspect (° from *N*)	**1.75 × 10** ^**−3**^ (4.94 × 10^−4^)	1.10 × 10^−3^ (8.73 × 10^−4^)	4.20 × 10^−4^ (3.91 × 10^−4^)	8.55 × 10^−4^ (6.39 × 10^−4^)
Annual Mean temperature (°C)	−2.09 × 10^−2^ (1.44 × 10^−2^)	**−5.18 × 10** ^**−2**^ (2.47 × 10^−2^)	−1.84 × 10^−2^ (1.16 × 10^−2^)	**−4.31 × 10** ^**−2**^ (1.90 × 10^−2^)
Annual Mean Precipitation (mm)	7.14 × 10^−4^ (5.25 × 10^−4^)	1.10 × 10^−3^ (9.37 × 10^−4^)	2.75 × 10^−4^ (4.08 × 10^−4^)	**1.45 × 10** ^**−3**^ (6.66 × 10^−4^)
Biomass (Mg ha^−1^)	**−1.37 × 10** ^**−3**^ (2.52 × 10^−4^)	**−1.04 × 10** ^**−3**^ (4.25 × 10^−4^)	−2.11 × 10^−4^ (1.72 × 10^−4^)	−3.14 × 10^−4^ (2.81 × 10^−4^)

*Note*: Results for the angiosperm only subset are reported in Table S2. Model *R* ^2^ is reported under each specific model (columns). Parameter estimates in bold represent a *p*‐value less than .05. Values in parenthesis under parameter estimates are the associated standard error.

Additionally, understory plant species richness was slightly positively associated with aspect for our all‐species metric and angiosperm only metric (Table 1; Table S2; *β* = .00175 and *β* = .00182, respectively), such that more northerly plots had on average higher species richness. The metric for species richness that only included species with associated functional trait data and understory species diversity did not covary with aspect. Elevation (m), mean annual temperature (°C) and mean annual precipitation (mm), were not statistically significant predictors of understory plant species richness or Simpson's diversity across plots, with two exceptions. Mean annual temperature was slightly and negatively related to species richness when using the metric that only included species with functional trait data (Table 1; *β* = −0.052). Mean annual temperature and annual mean precipitation were correlated with our smallest subset of species diversity (Table 1; *β* = −0.043 and *β* = .001, respectively).

We observed little differentiation between our two groups of plots (≥75th percentile aboveground live tree biomass carbon, <75th percentile aboveground live tree biomass carbon) in our NMDS ordination (Figure S2). Additionally, plots varied greatly within each group and there was a large overlap between the 95% confidence ellipsoids for the two groups (Figure S2). The results were not sensitive to the choice of threshold, which we tested at the 65th and 85th percentile as well (Figure S2).

**FIGURE 3:**
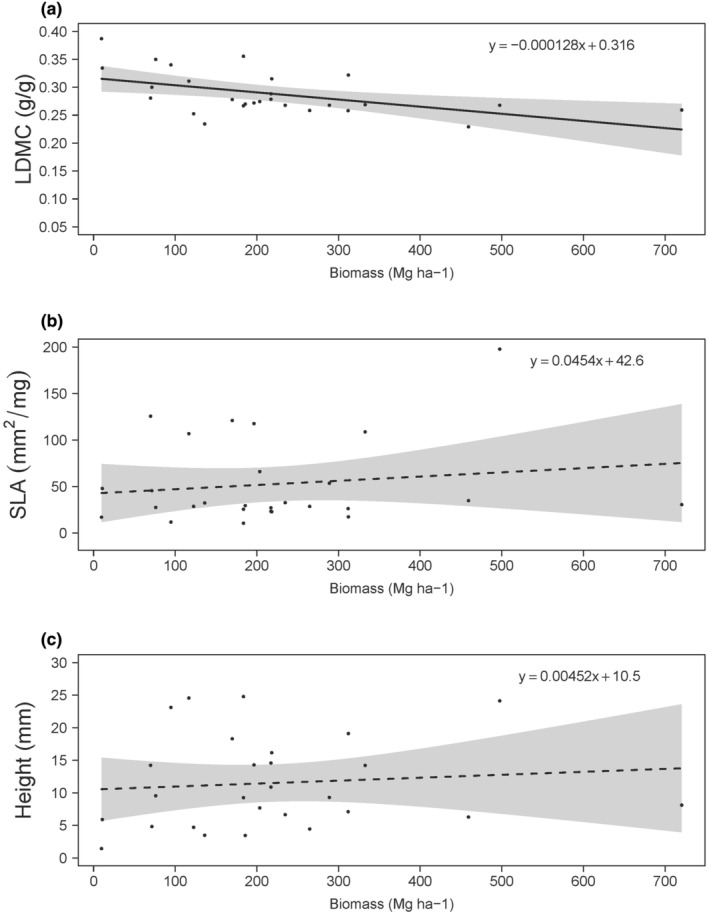
Correlations of community weighted mean trait values for leaf dry matter content (LDMC; a), specific leaf area (SLA; b), and height at maturity (c), with live tree biomass carbon (Mg ha^−1^). Dashed lines represent non‐significant correlations. Shaded regions represent 95% confidence intervals of slope for each panel. Model slope estimates and intercepts are reported in the top right corner.

The community weighted mean trait value of leaf dry matter content had a slight but negative correlation with the estimate of live tree biomass carbon (Figure 3; *β* = −0.0001). The variance of community weighted mean values for leaf dry matter content was small (0.229–0.387 g/g) compared to species level averages (0.076–0.508 g/g). The community weighted mean trait values for specific leaf area and plant height did not have statistically significant correlations with the estimate of aboveground live tree biomass carbon (Figure 3). The variance of species level averages for specific leaf area and plant height was larger than that of leaf dry matter content (3.95–208 mm^2^/mg and 0.041–27.8 mm, respectively) with correspondingly larger community weighted mean averages as well (10.6–198 mm^2^/mg and 1.44–24.8 mm for specific leaf area and height, respectively).

## 4 DISCUSSION

The tradeoffs between understory plant community biodiversity and aboveground live tree biomass carbon storage are understudied, even though understory plant species are taxonomically diverse (Enquist et al., 2019) and contribute to ecosystem services such as biogeochemical cycling (Speckman et al., 2015), wildlife habitat and nutrition (Springer et al., 2022), and carbon cycling (Chen et al., 2019). To elucidate the relationship of understory plant species richness and diversity to aboveground live tree biomass carbon storage, we leveraged existing FIA plot data in the Tongass NF along a gradient of high‐resolution modeled carbon data. Understory plant species richness, but not diversity as measured through the Simpson's diversity index, decreased significantly along a gradient of aboveground live tree biomass carbon, regardless of which grouping for species richness we used (Figure 2; Figure S1; Table 1; Table S2). The response of the understory plant community at the plot level suggests that the interrelation of understory plant biodiversity and aboveground live tree biomass carbon storage is not straightforward. Decreases in understory species richness could, therefore, reflect a tradeoff between the understory plant community and aboveground carbon storage. However, the absence of an effect of aboveground carbon storage on plant diversity indicates that tradeoffs may be more complicated or take place during the early stages of community assembly, potentially during overstory canopy closure as mediated through overstory leaf area, which would influence available light available in the understory in a system that is typically considered energy limited (Zangy et al., 2021). Community weighted mean leaf dry matter content was significantly lower in plots with high aboveground live tree biomass carbon storage suggesting slower growing plant strategies are less effective in the presence of high biomass carbon dense trees in the overstory.

We expected plot level plant species richness and diversity to have a tradeoff with overstory aboveground live tree biomass carbon storage (Kobayashi et al., 2023). Tradeoffs between understory species richness and diversity and overstory tree basal area are well documented in the context of disturbance ecology and have been observed following insect outbreaks (Carter et al., 2022; Pappas et al., 2020) and fire (Laughlin & Fulé, 2008; Stevens et al., 2019), but this has not been extended to carbon storage in intact forests. Understory plant species richness was on average lower in plots with high aboveground live tree biomass carbon storage, with the magnitude of effect depending on the group of species included (Figure 2a; Figure S1a; Table 1). Interestingly, we observed no effect of aboveground live tree biomass carbon storage on understory plant diversity using the Simpson's diversity index (Figure 2b; Figure S1b; Table 1). The variation in results using two methods to quantify understory plant biodiversity suggests trees that are biomass carbon dense may competitively exclude already rare understory species with narrow requirements for growth and survival, which would decrease species richness with minor consequences to associated plant species diversity. Our results largely align with previous research that observes smaller correlations between biodiversity and carbon at smaller spatial scales (Di Marco et al., 2018), although the direction of the correlation in this research is opposite from investigations on vertebrate diversity and tree carbon. This interaction can be indirectly mediated through environmental change caused by trees with high biomass carbon storage. In other words, rare species are lost as carbon stocks increase. The effect of aboveground live tree biomass carbon storage on understory plant biodiversity is complex and requires further elaboration.

The relationships of understory plant richness and diversity with environmental covariates for aspect, elevation, and temperature were mostly statistically insignificant despite the relationship these variables have on tree biomass carbon storage (Buma & Barrett, 2015; Carter & Buma, 2024), and the observed effect of tree biomass carbon storage on understory plant species richness. We did observe a statistically significant effect of aspect on species richness, such that more northerly aspects were associated with higher species richness. More northerly aspects may have a higher number of understory plant species on average because northerly aspects typically have lower average tree carbon storage due to energy limitations (Buma & Barrett, 2015).

We anticipated large trees with high aboveground biomass carbon to be highly competitive for resources that promote aboveground biomass carbon storage and limit growth and survival for understory plant species, such as light or nutrients. Thus, the resource acquisition by large, high‐carbon trees likely interferes with resource availability for understory plant species, which may be reflected by their associated functional traits (Laughlin et al., 2020). We detected a negative correlation of community weighted mean leaf dry matter content and aboveground live tree biomass carbon storage (Figure 3), which suggests that communities in the presence of large and competitive trees had on average trait values associated with a less conservative growth strategy. Species present in these communities might need to rapidly take advantage of available nutrients (Westoby et al., 2002), which would be indicative of an acquisitive growth strategy. The competition for nutrients imposed by large trees may constrain the distribution of trait values at the community level for leaf dry matter trait content, as gradients along a selective pressure (e.g., precipitation) have been observed to limit the distributions of corresponding functional trait values (Dwyer & Laughlin, 2017). Other community weighted mean trait values did not correlate to the gradient of aboveground live tree biomass carbon storage and had comparatively more community level variation in trait values. This variation could be attributed to several factors. First, the variation may be due to an insufficient sample size capable of detecting a trend in other community level strategies. A post‐hoc power analysis provides evidence that our sample size was only sufficient to detect an effect as small as 0.577 (*df* = 25, *n* = 27, *α* = .05). Second, high variation could be present because there is not sufficient selective pressure for these trait values, and natural random variation exists. Third, there may be a variety of strategies with multiple traits involved in the understory plant community to grow and survive in the presence of competition for light and nutrients (Díaz et al., 2016; Dwyer & Laughlin, 2017), which would not be detectable with our analysis. The lack of functional trait data for rare species that were competitively excluded precludes further investigation on trait values not present in high carbon plots. Traits that did not have adequate representation in the TRY database, such as dispersal‐related traits or belowground traits, may represent some of the varied strategies that confer success (Bergmann et al., 2020; Westoby, 1998). Fourth, aboveground carbon storage may not adequately represent all mechanisms for resource competition between the overstory and understory plant community. Overstory leaf area likely drives competitive interactions for light resources and may peak at early stages of stand development then remain relatively stable while trees accumulate carbon (Rago et al., 2021). Regardless, the strategies employed by the understory plant community to grow and persist under large and competitive trees need further elucidation with larger sample sizes and a more complete selection of traits both for the overstory and understory plant community not readily available from global trait databases.

### 4.1 Limitations

Our conclusions are limited by the data available through the FIA dataset and associated functional trait data that are publicly available. Despite having high‐resolution modeled carbon data for the entire forest (Carter & Buma, 2024), only 27 of the 1388 FIA plots (2%) within the Tongass NF have associated understory plant community data (Gray et al., 2012). We were able to detect an effect of aboveground live tree biomass carbon storage on understory species richness, regardless of which metric of richness we used (Figure 2a). However, we were unable to detect a similar effect on understory species diversity as measured by the Simpson diversity index (Figure 2b), which may change if more understory data are collected for FIA plots. Additionally, we were only able to access functional trait data for 69 of the 230 species present in the dataset. These 69 species were locally important, representing 24% of the total understory plant species cover, but estimates would be improved with additional functional trait data. Importantly, the dataset does not include non‐vascular understory plant species. This is significant because non‐vascular species comprise up to 25% of understory plant biomass across this region (Den Ouden & Alaback, 1996). Non‐vascular species play a role in nitrogen fixation (Weber & Vancleve, 1981), which would alter the strategies neighboring vascular plants employ.

## 5 CONCLUSIONS

Maintaining biodiversity in a rapidly changing world requires carefully devised management strategies. We must examine the trade‐offs in managing forested landscapes for both carbon storage and old growth and impacts on understory plant biodiversity, given that optimizing one goal may limit the other. For example, primarily focusing on carbon management may lead to deleterious effects on plant biodiversity, understory forage for wildlife, or other impacts. Working in Tongass NF, we found on average a decline of 2 understory plant species for every increase in 50 Mg ha^−1^ of tree biomass carbon, although slightly more losses occur at early levels of biomass accumulation. The response of understory plant community biodiversity to overstory competition is not straightforward, and the specific mechanisms that control the response need further elucidation in future research.

## AUTHOR CONTRIBUTIONS

**Trevor A. Carter:** Conceptualization (lead); data curation (lead); formal analysis (lead); investigation (lead); methodology (lead); validation (lead); visualization (lead); writing – original draft (lead). **Brian Buma:** Conceptualization (supporting); funding acquisition (lead); project administration (lead); supervision (lead); writing – review and editing (lead).

## CONFLICT OF INTEREST STATEMENT

The authors declare that there are no competing interests regarding the publication of this article.

## Supporting information


Data S1.

## Data Availability

Forest Inventory and Analysis data and plant functional trait data are available publicly through the USFS [https://apps.fs.usda.gov/fia/datamart/datamart.html] and TRY [https://www.try‐db.org/TryWeb/Home.php] database respectively. Estimates of aboveground live tree biomass carbon are available on Dryad [https://doi.org/10.5061/dryad.3tx95x6nn]. Corresponding code is available on Github  https://github.com/TrevorACarter/Carter‐Buma2024b.
