# Supplementary material for: Understory plant biodiversity is inversely related to carbon storage in a high carbon ecosystem
Source: Ecol Evol. 2024 Oct 27;14(10):e70095. doi: 10.1002/ece3.70095 (PMC11512734; doi:10.1002/ece3.70095)
Supplement: Supplementary file 1 — Data S1. [file ECE3-14-e70095-s001.docx]

**Supplemental Figures**

Table S1. List of 115 species with associated USDA species codes, average species trait values for LDMC, SLA, and vegetative height, and whether petiole was included for SLA measurements.

Table S2. Parameter estimates (rows) for the effect of the median estimate of aboveground live tree biomass carbon and other known important climatic and topographic predictors on understory species richness and diversity for each of the subsets of species inclusion. Model R^2^ are reported under each specific model (columns). Parameter estimates in bold represent a p-value less than 0.05. Values in parenthesis under parameter estimates are the associated standard error.

| Parameter | all spp. richness  (*R^2^ = 0.325*) | angiosperm spp. richness  (*R^2^* *= 0.344*) | spp. with trait data richness  (*R^2^* *= 0.205*) | all spp. diversity  (*R^2^* *= 0.240*) | angiosperm spp. diversity  (*R^2^* *= 0.187*) | spp. with trait data diversity  (*R^2^* *= 0.283*) |
| --- | --- | --- | --- | --- | --- | --- |
| Intercept | **3.54**  (1.71 × 10^-1^) | **3.35**  (1.97 × 10^-1^) | **2.39**  (3.03 × 10^-1^) | **7.48 ×** **10^-1^**  (1.30 × 10^-1^) | **7.22 ×** **10^-1^**  (1.51 × 10^-1^) | 2.89 × 10^-1^  (2.12 × 10^-1^) |
| Elevation (m) | 1.63 × 10^-4^  (1.77 × 10^-4^) | 2.07 × 10^-4^  (2.01 × 10^-4^) | -4.50 × 10^-4^  (3.30 × 10^-4^) | 1.12 × 10^-5^  (1.43 × 10^-4^) | 6.82 × 10^-5^  (1.66 × 10^-4^) | -2.07 × 10^-4^  (2.33 × 10^-4^) |
| Aspect (° from N) | **1.75 × 10^-3^**  (4.94 × 10^-4^) | **1.82 ×** **10^-3^**  (5.72 × 10^-4^) | 1.10 × 10^-3^  (8.73 × 10^-4^) | 4.20 × 10^-4^  (3.91 × 10^-4^) | 4.10 × 10^-4^  (4.56 × 10^-4^) | 8.55 × 10^-4^  (6.39 × 10^-4^) |
| Annual Mean temperature (°C) | -2.09 × 10^-2^  (1.44 × 10^-2^) | -2.82 × 10^-2^  (1.61 × 10^-2^) | **-5.18 ×** **10^-2^**  (2.47 × 10^-2^) | -1.84 × 10^-2^  (1.16 × 10^-2^) | -1.74 × 10^-2^  (1.35 × 10^-2^) | **-4.31 ×** **10^-2^**  (1.90 × 10^-2^) |
| Annual Mean Precipitation (mm) | 7.14 × 10^-4^  (5.25 × 10^-4^) | 4.69 × 10^-4^  (6.03 × 10^-4^) | 1.10 × 10^-3^  (9.37 × 10^-4^) | 2.75 × 10^-4^  (4.08 × 10^-4^) | 1.99 × 10^-4^  (4.75 × 10^-4^) | **1.45 ×** **10^-3^**  (6.66 × 10^-4^) |
| Biomass (Mg ha^-1^) | **-1.37 ×** **10^-3^**  (2.52 × 10^-4^) | **-1.66 ×** **10^-3^**  (3.03 × 10^-4^) | **-1.04 ×** **10^-3^**  (4.25 × 10^-4^) | -2.11 × 10^-4^  (1.72 × 10^-4^) | -1.40 × 10^-4^  (2.00 × 10^-4^) | -3.14 × 10^-4^  (2.81 × 10^-4^) |


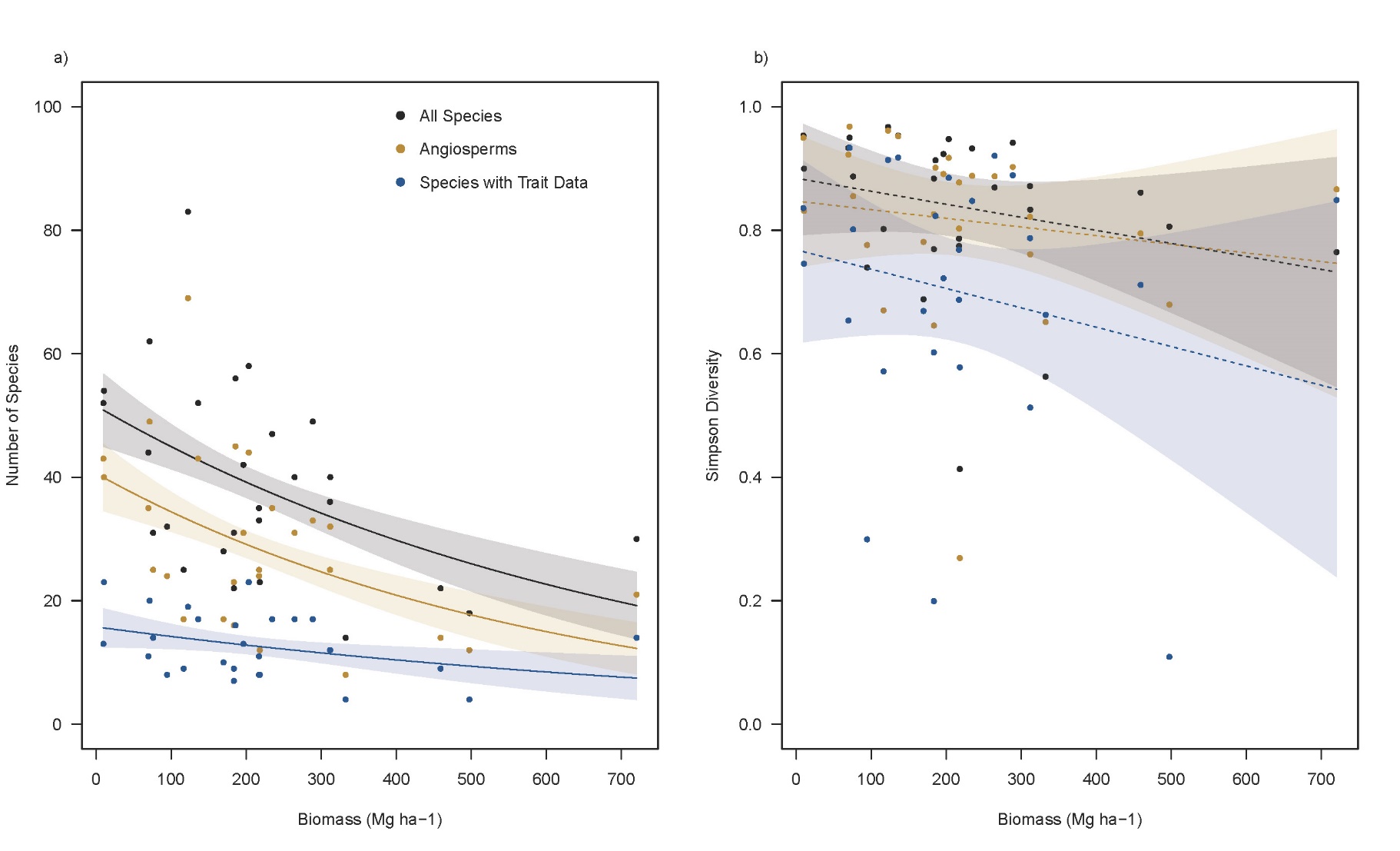
Figure S1. Represents the relationship of species richness (a) and Simpson diversity index (b) as a function of aboveground live tree biomass (Mg of Carbon ha^-1^). Solid lines represent statistically significant (alpha = 0.05) best fit regression lines from our models, while dashed lines represent statistically non-significant relationships. The shaded polygons represent the 95% confidence interval around the beta estimate for each corresponding slope (see supplemental materials). The estimated partial effect of live tree biomass carbon on all species found in the understory (black line; 100% of total understory cover), only angiosperms which excludes ferns, club mosses, and gymnosperms (gold line; 56% of total understory cover), species with associated trait data (blue line; 23% of total understory cover).


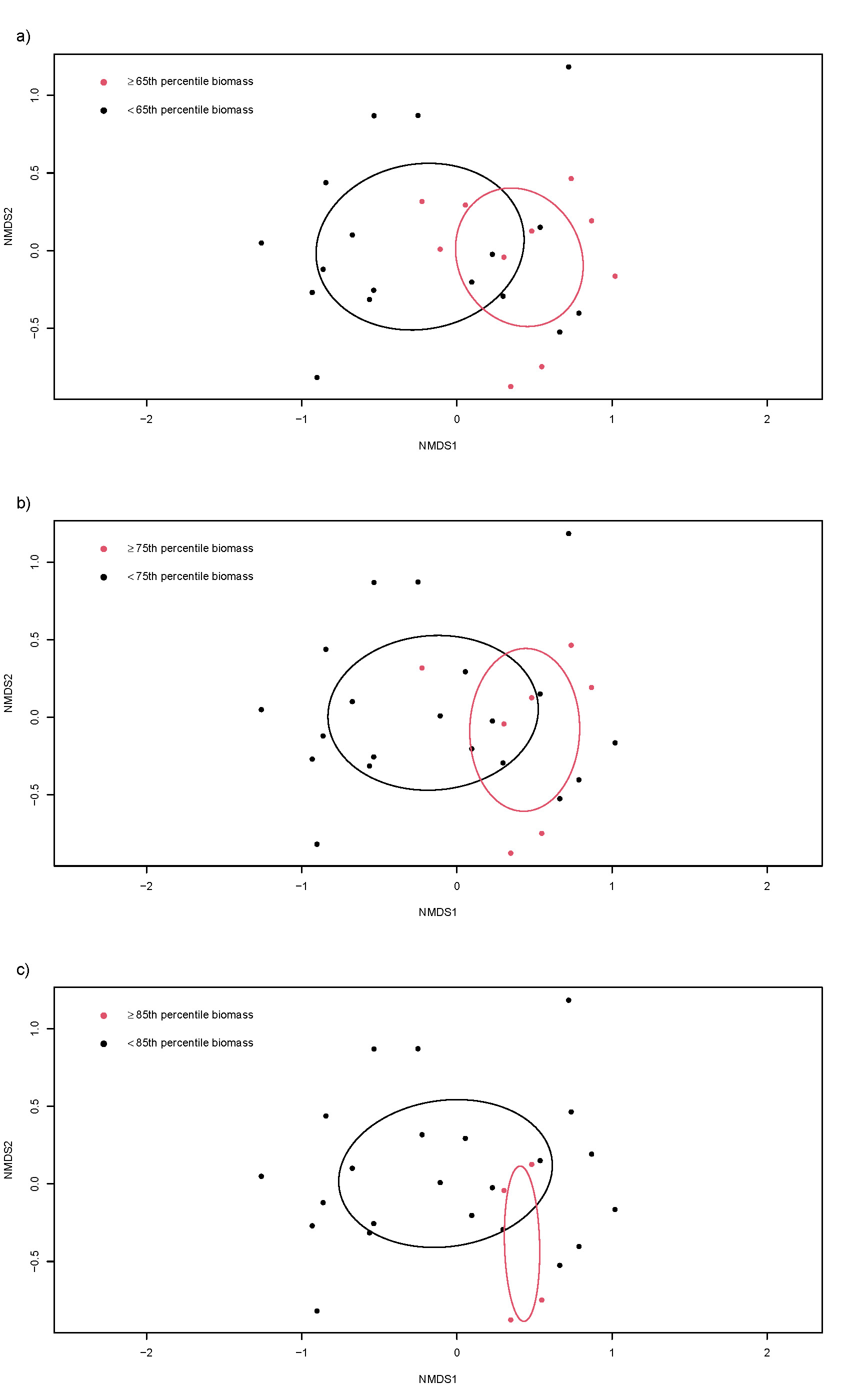


Figure S2. Non-metric dimensional scaling (NMDS) ordination with plant communities found in areas with greater than or equal to the (a) 65^th^ percentile tree biomass carbon and less than the 65^th^ percentile tree biomass carbon, (b) 75^th^ percentile tree biomass carbon and less than the 75^th^ percentile tree biomass carbon, (c) 85^th^ percentile tree biomass carbon and less than the 85^th^ percentile tree biomass carbon. The ellipses represent the 95% confidence interval for each respective group.
